# P05-07 The association between 24-hour activity composition and back pain in Slovenian university students

**DOI:** 10.1093/eurpub/ckac095.074

**Published:** 2022-08-29

**Authors:** Kaja Kastelic, Michael D Burnard, Nejc Šarabon, Nastja Podrekar, Željko Pedišić

**Affiliations:** 1 Department of Health Study, Andrej Marušič Institute, University of Primorska, Koper, Slovenia; 2 S2P, Science to Practice, Ltd., Ljubljana, Slovenia; 3 Human Health in the Built Environment, InnoRenew CoE, Izola, Slovenia; 4 Department of Technology, Andrej Marušič Institute, University of Primorska, Koper, Slovenia; 5 Faculty of Health Sciences, University of Primorska, Izola, Slovenia; 6 Institute for Health and Sport, Victoria University, Melbourne, Victoria, Australia

**Keywords:** compositional data analysis, time use, spinal health, risk factor, epidemiology

## Abstract

**Background:**

Back pain is the most common musculoskeletal symptom. Several risk/protective factors, including sedentary behaviour, physical activity and sleep, have been proposed. Research has typically examined these time-use behaviours in isolation, ignoring the compositional nature of time-use data. The aim of this study was to determine the relationship between a 24-hour activity composition and back pain in university students using compositional isotemporal substitution modelling.

**Methods:**

A cross-sectional study of 135 Slovenian university students (20 ± 2 years, 70% male) assessed 24-hour time use and back pain. Volunteers completed the SIMPAQ questionnaire (asking about the activity time divided into three categories: sedentary, in bed, and physical activity) and the BackPEI questionnaire (asking about back pain in the past 3 months). The compositional isotemporal substitution analysis based on a logistic regression model was used to examine the association between the activity composition and the occurrence of back pain. The compositional isotemporal substitution analysis based on a linear regression model was used to examine the association between the activity composition and back pain intensity, for those that experienced it. Both models were adjusted for age, sex and BMI.

**Results:**

The prevalence of back pain in the past 3 months was 62%. The 24-hour activity composition was associated with back pain intensity in the symptomatic subgroup, while no associations with the occurrence of back pain was found. Reallocation of 30 minutes from sedentary behaviour to physical activity was associated with a mean reduction of back pain intensity by 0.1 (95% CI: 0.01 to 0.201) on a continuous 10-point Visual Analog Scale. Likewise, the opposite reallocation was associated with an increase of back pain intensity by 0.1 (95% CI: 0.003 to 0.21). No significant associations with the intensity of back pain were found for reallocations of 30 minutes to and from bed time.

**Conclusion:**

Study findings indicate that reallocating time from sedentary behaviour to physical activity has a favourable association with back pain intensity. However, the effect size was relatively small and findings need to be interpreted with caution. Further studies including more precise measures of exposure and with larger sample sizes are warranted

